# The Function and Regulation of SAPCD2 in Physiological and Oncogenic Processes

**DOI:** 10.7150/jca.65949

**Published:** 2022-04-18

**Authors:** Amy L. Baker, Liqin Du

**Affiliations:** Department of Chemistry and Biochemistry, Texas State University, 601 University Drive, San Marcos, TX, 78666, USA.

**Keywords:** p42.3, SAPCD2, Cell Cycle, Cell Fate, Cancer, Metastasis.

## Abstract

The Suppressor APC Domain Containing 2 (SAPCD2) gene, also known by its aliases p42.3 and c9orf140, encodes a protein with an approximate molecular weight of 42.3 kDa. It was initially recognized as a cell cycle-associated protein involved in mitotic progression. Since the initial discovery of this gene, emerging evidence has suggested that its functions extend beyond that of regulating cell cycle progression to include modulation of planar polarization of cell progenitors and determination of cell fate throughout embryonic development. The underlying mechanisms driving such functions have been partially elucidated. However, the detailed mechanisms of action remain to be further characterized. The expression level of SAPCD2 is high throughout embryogenesis but is generally absent in healthy postnatal tissues, with restored expression in adult tissues being associated with various disease states. The pathological consequences of its aberrant expression have been investigated, most notably in the development of several types of cancers. The role of SAPCD2 in tumorigenesis has been supported by *in vitro*, *in vivo*, and retrospective clinical investigations and the mechanisms underlying its oncogenic function have been partially revealed. The potential of SAPCD2 as a diagnostic marker and therapeutic target of cancers have also been explored and have shown great promise. However, many questions pertaining to its oncogenic mechanisms as well as its value as a diagnostic marker and therapeutic target remain to be answered. In addition to its function as an oncogene, an involvement of SAPCD2 in other pathological processes such as inflammation has also been implicated and provides additional directions that warrant future investigation. This article reviews the current understanding of the normal cellular functions of SAPCD2 and the relevance of SAPCD2 in disease development with a primary focus on tumorigenesis. The mechanisms that regulate p43.2 expression, including the potential role of microRNAs in regulating its expression, are also reviewed. To the best of our knowledge, we are the first to comprehensively review the published findings regarding the physiological and pathological functions of this gene.

## Background

The SAPCD2 gene (NCBI Gene ID 89958) is highly expressed in embryonic cells in a cell cycle-dependent manner. It is located on the long arm of chromosome 9 at position 34.3 in the human genome 1. It encodes a 389-amino acid (aa) protein product with an estimated molecular weight of 42.3 kDa, inspiring one of its common alias p42.3 1. The SAPCD2 protein was first isolated and characterized in 2007 by Xu *et al*. when screening for differentially expressed proteins between the G_1_ and S phases of the cell cycle in the human gastric carcinoma (GC) cell line BGC823 1. Although the normal biological functions of SAPCD2 are not fully understood, current evidence supports an important role of SAPCD2 in determining the specific direction of cell differentiation throughout early fetal development, where it regulates the spatial orientation of cellular scaffolds, directs cell polarity during mitosis, and controls cell fate decisions.

While its expression is normally low or absent in healthy postnatal tissues, restored expression in adult tissues is highly associated with various disease states. The potential contributions of its aberrant expression to disease development, particularly tumorigenesis, are increasingly recognized. Emerging evidence has supported that the expression level of SAPCD2 may be a useful prognostic indicator of several different types of cancers, including gastric cancer, colorectal cancer, hepatocellular carcinoma, melanoma, renal cell carcinoma, nasopharyngeal carcinoma (NPC), lung cancer, and breast cancer. The mechanisms underlying its oncogenic function have also been partially revealed.

Despite the numerous novel findings on SAPCD2 documented in peer-reviewed research papers, only one literature review on this gene has been published 2. In this article published in 2015, Cao *et al*. focused on reviewing the function of SAPCD2 in regulating the cell cycle progression 2. Given the vast knowledge on its diverse physiological and pathological functions that has been gained in recent years, a more comprehensive review is needed to facilitate knowledge sharing on this important gene in the biomedical community, which motivated us to write this review article. We first review the SAPCD2 gene, its mRNA transcript and protein product, and the current understanding of the normal cellular functions of the SAPCD2 protein. Following this is a discussion of the recent findings on its role in pathological processes with an emphasis on its role in tumorigenesis. The findings on its potential diagnostic and therapeutic values in human malignancies are also reviewed. Finally, we review the mechanisms that regulate its expression, including the potential role of microRNAs (miRNAs) in regulating SAPCD2 expression levels. We hope that this summative report will provide researchers with a comprehensive understanding of the importance of this gene in normal cellular processes and its relevance to diseases and will promote further investigations into the development of novel diagnostic and therapeutic tools.

## 1. The SAPCD2 Gene, Its Transcript and Its Protein Product

**Figure 1:**
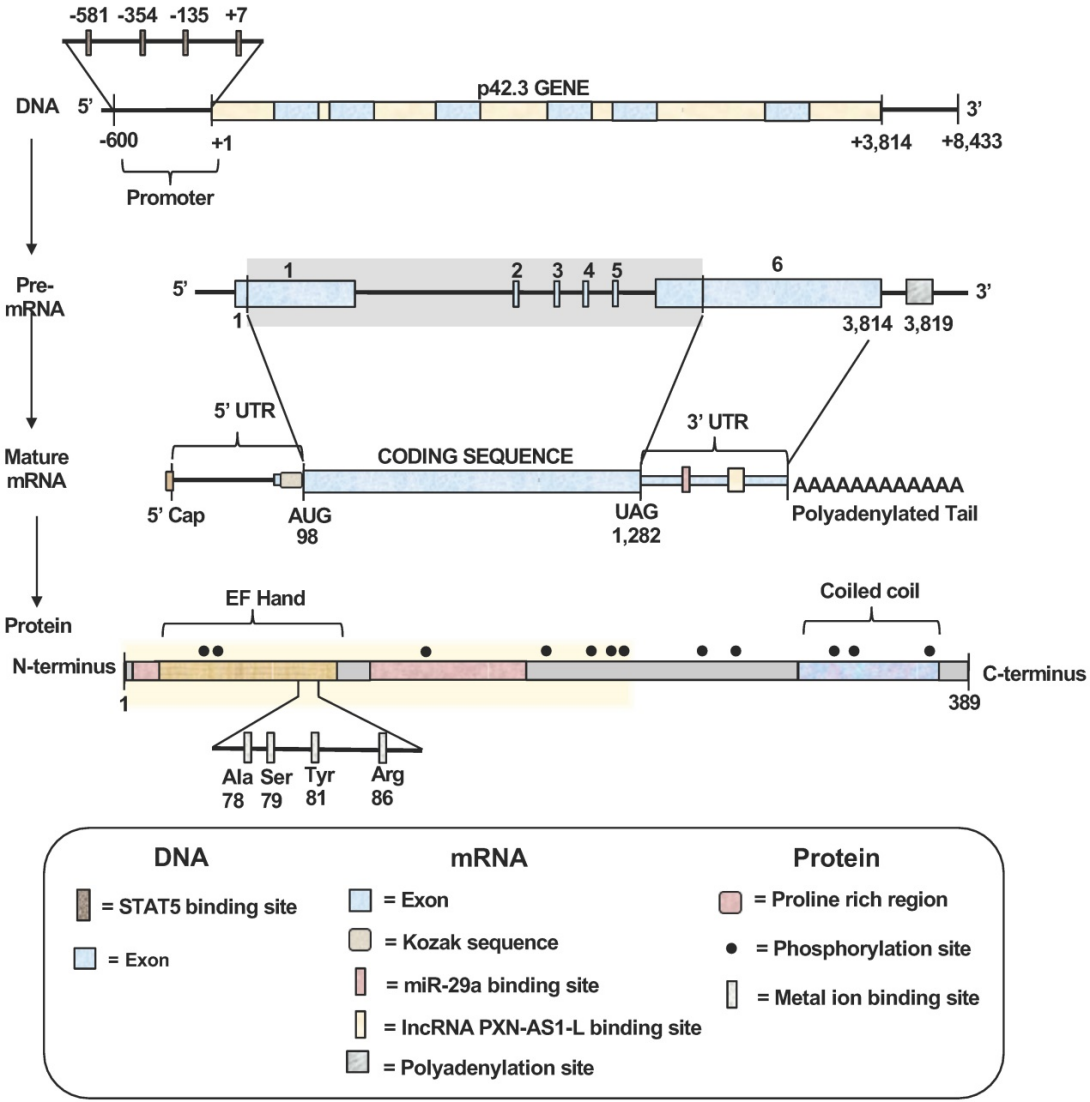
** Schematic structures of SAPCD2 gene, mRNA and protein. (1)** Gene structure. The SAPCD2 gene is 3,814 nucleotides (nts) long. The promoter region contains four validated STAT5 binding sites. **(2)** mRNA structures. Splicing of the pre-mRNA transcript generate the mature mRNA transcript with protein coding region of 1,184 nts. A Kozak consensus sequence exists in the upstream of the coding region and serves as the translation initiation site. Portions of the exons 1 and 6 serve as the 5'UTRs and 3'UTRs, respectively, in the mature mRNA. The 3'UTR contains predicted targets sites of multiple miRNAs with the target site of miR-29a binding site having been validated. The 3' UTR also contains a validated lncRNA-PXN-AS1-L binding site. **(3)** Protein structure. The final translation product is a protein (389 aa) containing an EF-Hand domain at the N-terminus with a Coiled Coil domain at the C-terminus and two proline-rich regions. Twelve putative phosphorylation sites exist at positions 26, 29, 128, 181, 211, 217, 219, 279, 284, 350, 357, and 377 of the protein sequence. A putative metal ion-binding site involving four amino acids (Ala78, Ser 79, Tyr 81 and Arg 88) within the EF-hand domain is predicted. The highlighted region spans amino acids 1-239 and represents the region of the SAPCD2 protein that is essential for interaction with AXIN1.

To date, full characterization of the gene, mRNA transcript and protein structures of SAPCD2 has not been completed. However, certain characteristics have been identified, as summarized in **Figure 1.** The SAPCD2 gene is 3,814 nucleotides (nts) long and contains 6 exons. To date, information regarding the regulatory elements upstream or downstream of the SAPCD2 gene sequence is not fully defined. However, some progress has been made in identifying several regulatory elements that are fundamental to SAPCD2 transcriptional and translational processing. Four binding sites for the transcription factor STAT5 have been identified within the first 600 nts of the promoter region, and it was found that phosphorylation of STAT5 (pSTAT5) induced the transcriptional activation of SAPCD2. Furthermore, mutation of the four STAT5 binding sites together resulted in a significant reduction of their transcriptional activity as measured by luciferase activity 3. The physical interaction between pSTAT5 and its binding sites was revealed by Chromatin immunoprecipitation (ChIP) assay. In addition, EZH2 and β-catenin were detected in the ChIP immunoprecipitates, suggesting that the pSTAT5-mediated transactivation of SAPCD2 requires recruitment of EZH2 and β-catenin to the transcription complex 3.

The mature mRNA transcript of SAPCD2 contains a coding sequence (CDS) spanning 1,184 nucleotides in length, a 5'UTR of 97 nucleotides, and a 3'UTR of 2,532 nucleotides. A Kozak consensus sequence exists in the upstream region immediately preceding the start codon and serves as the translation initiation site. Portions of exons 1 and 6 encode regions of the 5'UTRs and 3'UTRs, respectively. The 3'UTR contains validated targets site of miRNA miR-29a (seed positions 213-219) 4. Additionally, The 3'UTR also contains a validated binding site for long non-coding RNA (lncRNA) PXN-AS1-L at nt positions 387 - 484 5. Both miR-29a and PXN-AS1-L have been shown to modulate SAPCD2 expression at the mRNA and protein levels, where miR-29a reduces expression levels and PXN-AS1-L enhances expression levels 4, 5.

Although the complete protein structure of SAPCD2 has yet to be illustrated, some significant features have been identified. The N-terminus of SAPCD2 contains an EF-hand motif spanning amino acids 7-87 1. The EF-hand motif is characterized by a helix-loop-helix structure containing a calcium-binding chelate ring flanked by two alpha helices 1, 6. The SAPCD2 DNA sequence encoding this motif is highly conserved in vertebrates, suggesting that the EF-hand motif may fulfill a critical role in the function of SAPCD2 7. EF-hand motifs are widely known to be associated with calcium-binding functions 8, and Hao *et al*. generated a 3D ligand binding model of the SAPCD2 EF-hand domain and predicted a metal ion binding site involving four amino acids within this region of SAPCD2 9. However, the physical interaction of calcium with SAPCD2 in this region requires experimental validation. The functional importance of EF-hand motifs has been recognized by their association with the calcium-binding S100 oncoprotein family, which consists of at least 25 members involved in a variety of important intracellular regulatory functions including proliferation, neoplastic transformation, differentiation, protein phosphorylation, inflammation, and maintenance of intracellular calcium homeostasis 8, 10, 11. Deregulation of calcium-mediated cell signaling has been associated with the promotion of specific oncogenic events, such as angiogenesis, metastasis, and tumor formation, and the potential therapeutic benefit of targeting calcium signaling as a valuable anti-cancer strategy is increasingly recognized 12. Altogether, the presence of an EF-hand motif within the SAPCD2 protein, along with the functional roles associated with this motif, may partially explain the importance of p42.3 in tumorigenesis as reviewed in later sections of this article. However, definitive functions of the EF-hand domain within the SAPCD2 protein have not been elucidated.

The C-terminus of SAPCD2 is characterized by a coiled-coil (CC) domain (aa 331-379), which may be responsible for the interactions between SAPCD2 and other proteins 1. The specific role that the CC domain plays in the function of the SAPCD2 protein, however, is poorly understood and requires further investigation. The SAPCD2 protein also includes two proline-rich regions (aa 8-14 and 97-169), which may also serve the function of mediating protein-protein interactions 1.

In the SAPCD2 amino acid sequence, multiple phosphorylation sites for checkpoint kinase 2 (Chk2), protein kinase C (PKC), and some important second messengers such as cAMP and cGMP have been predicted 1. However, the role played by these phosphorylation sites in regulating the activity of SAPCD2 remains largely undefined. Generally, the serine/threonine protein kinase Chk2 plays a key role in regulating cell cycle checkpoint arrest through the phosphorylation of several mitotic phase-inducing cell division cycle (CDC) phosphatases, inhibiting their activity and stalling mitotic progression. PKC is a family of 10 structurally related isozymes organized into three subfamilies (classical, novel, and atypical) with functional diversity. Although the direct role of PKC in oncogenesis is poorly defined, the oncogenic potential of some PKC-mediated processes has been demonstrated 13, 14. The presence of potential phosphorylation sites for Chk2 and PKC within SAPCD2 strongly suggests that SAPCD2 is a key downstream target of Chk2 and PKC and may be involved in a variety of Chk2- and PKC-mediated physiological or pathological processes.

## 2. The Cellular Functions of SAPCD2

**Figure 2:**
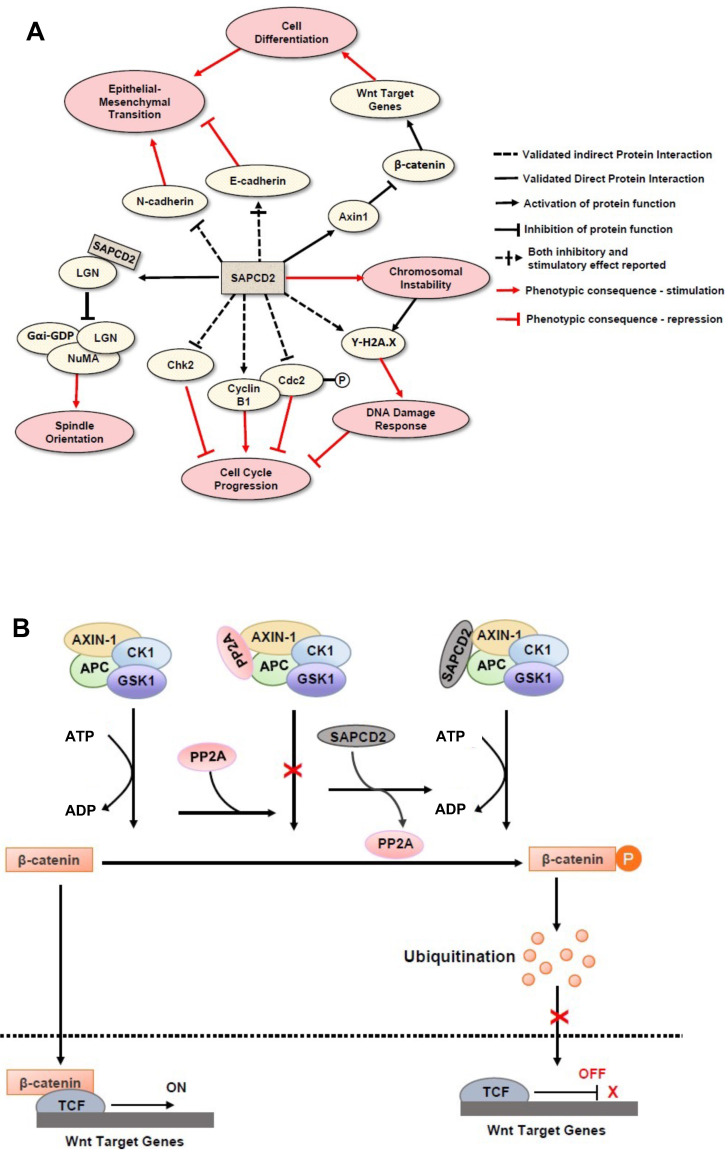
** Mechanisms of action of SAPCD2 protein in modulating normal and pathological cellular processes.** (A) The function of SAPCD2 as a modulator of cellular processes. Up to date, SAPCD2 is found to function as an important modulator multiple cellular processes, including cell cycle progression, spindle orientation, epithelial mesenchymal transition, chromosomal instability, DNA damage responses, and cell differentiation. Such functions of SAPCD2 determine the important role of SAPCD2 in the physiological and pathological processes, such as embryonic development, oncogenesis and inflammation. Some of the pathways have been validated in *in vitro* or *in vivo* studies, while others are predicted have not been experimentally validated sufficiently. The net effect of SAPCD2 on a given cellular process can be thought of as the product of interactions with each individual protein, such that two adjacent inhibitory interactions indicate a net positive effect, while adjacent inhibitory and stimulatory interactions indicate a net negative effect. **(B)** The role of SAPCD2 in modulating the Wnt/β-catenin signaling.

Initially, SAPCD2 was identified in a GC cell line as a protein with mitotic phase-dependent expression patterns and with depletion resulting in G_2_/M phase arrest 1. Based on these findings, it was predicted that the primary cellular function of the SAPCD2 protein is to participate in G_2_/M phase progression, and that its interactive molecular partners would be mitotic proteins. However, later studies indicated that SAPCD2 participates in a variety of protein interaction networks in addition to those involved in cell cycle progression, suggesting its involvement in a diverse range of physiological and pathological processes. Indeed, its involvement in a variety of normal and pathological cellular processes has been observed in studies from several research groups. The mechanisms of actions underlying its such functions were investigated and important findings are summarized in **Figure 2**.

### 2.1. Role of SAPCD2 in regulating cell cycle progression

Several studies have investigated the differential expression patterns of SAPCD2 throughout the cell cycle and the effect of manipulating SAPCD2 expression level on cell cycle distribution. Xu *et al.* initially reported that SAPCD2 expression level in G_1_ and M phases was higher than in S and G_2_ phases, with peak expression levels in the M phase compared to the other three phases in human gastric cancer (GC) cell lines 1. This group further found that depletion of SAPCD2 expression arrested cells in the G_2_/M phase, and SAPCD2-depleted GC cells exhibited notable morphological changes including enlargement, abnormal multinucleation, and pseudopodia, indicating that they were unable to progress to division as normal 1. Mao *et al.* reported that SAPCD2 expression level peaked in the early G_1_ phase, dramatically decreased in late G_1_, S, and G_2_ phases, and increased again during the M and G_2_ phases in gastric, breast, and lung cancer cell lines 15. These observations in general are consistent with what Xu *et al.* reported, although there is an inconsistency regarding the peak expression level of SAPCD2 between G_1_ and M phases. Despite this inconsistency, Mao *et al.* observed that stable silencing of SAPCD2 in the human GC cell line BGC823 leads to an increased accumulation of cells in the G_2_/M phases 15, consistent with observations with Xu *et al.* Mao *et al.* further found that exogenous overexpression of SAPCD2 in a normal embryonic fibroblast cell line led to a decreased accumulation of cells in the G_2_/M phase 15. Zhang *et al.* investigated the differential expression of SAPCD2 in a panel of twelve human GC cell lines and found that 9 out of 12 GC cell lines expressed SAPCD2 16. By observing the cell cycle progression of the human GC cell line BGC823 synchronized at the G_1_/S phase border by Nocodazole, this group found that SAPCD2 protein level peaked in the early G_1_ phase, declined throughout G_1_ progression, and increased again during G_2_/M phase progression 16. The differential expression pattern of SAPCD2 in different cell cycle phases in the BGC823 cell line is consistent with observations made by Mao *et al.* as reviewed above 15. Together, these results indicate that SAPCD2 expression stimulates accelerated mitotic progression*.* However, Luo *et al.* later observed that overexpressing SAPCD2 led to a slightly increased accumulation of cells in the G_2_/M phase within the poorly-differentiated colorectal carcinoma (CRC) cell line RKO 17. The same group found that the effect of SAPCD2 knockdown on cell cycle redistribution differed between two different CRC cell lines, where knockdown of SAPCD2 led to a peak accumulation of cells in the G_1_ phase and decrease accumulation in S phase in the RKO cell line but a decreased accumulation of cells in the G_1_ phase with an increase in S phase in the HCT116 cell line 17. Another study showed that knockdown of SAPCD2 in a GC cell line also led to cell cycle arrest at the G_1_ phase 4. Altogether, these conflicting observations suggest that the role of SAPCD2 expression on cell cycle distribution is complex and likely cell type specific.

SAPCD2 likely regulates cell cycle progression by interacting with multiple key pathways involved in cell cycle control. In the study by Xu *et al.*, knockdown of SAPCD2 in GC cells resulted in an increased expression of Chk2 and a decreased expression of cyclin B1 1, which are two key proteins involved in M-phase regulation. Mao *et al.* demonstrated in NIH3T3 cells that SAPCD2 modulates the Cyclin B1/Cdc2 complex, a complex that serves to promote mitotic phase entry and progression 15. Specifically, they demonstrated that SAPCD2 overexpression upregulates Cyclin B1 expression and decrease the phosphorylation of Cdc2 at the Tyr15 position (Cdc2--Tyr15). This phosphorylation event would normally inactivate Cdc2 and repress mitosis; however, the overexpression of SAPCD2 disrupts this process and ultimately promotes mitotic entry. The correlation between the expression levels of SAPCD2 and this important cell cycle modulator provides an additional mechanism through which SAPCD2 enhances mitotic progression 15.

### 2.2. Role of SAPCD2 in regulating cell fate and polarization

Evidence has suggested that SAPCD2 functions as a key modulator of various cellular processes important to determine cell fate during normal embryonic development, such as cell polarization, differentiation, and apoptosis. These functions may enable SAPCD2 to contribute to tumorigenesis and development-associated diseases by retaining stem cell-like characteristics when re-activated beyond the normal developmental processes. As presented above, SAPCD2 plays an important role in regulating cell cycle progression. Because the cell fate determination process is closely coupled with cell cycle redistribution, the expansive involvement of SAPCD2 in regulating cell cycle progression explains, at least partially, its role in regulating cell fate. Recently, SAPCD2 was found to modulate cell fate through additional molecular mechanisms as reviewed below.

In an investigation aimed at elucidating the mechanisms controlling spindle orientation in retinal progenitor cells (RPCs), a key event during the development of vertebrate retina, Chiu *et al.* revealed that SAPCD2 functions as a negative regulator of the Gαi-LGN-NuMA complex by interacting with several tight junction and polarity proteins including the LGN protein (named based on the 10 Leucine-Glycine-Asparagine repeats in its amino acid sequence), also called G Protein Signaling Modulator 2 (GPSM2)) 18. The balance between horizontal and vertical terminal cell divisions in RPCs is essential for normal retinal development. Horizontally dividing RPCs produce two photoreceptor cell progenies and are considered to be symmetrical, while vertically dividing RPCs produce one photoreceptor cell and one other cell type (bipolar, amacrine, or M
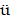
ller glial cell) and are considered to be asymmetrical 18. The LGN protein plays a key role in regulating mitotic spindle orientation by anchoring to the apical domain of the plasma membrane through attachment to Gαi and recruits NuMA to localize along the apico-basal axis, ultimately promoting vertical cell divisions 18. The N-terminal tetratricopeptide repeat (TPR) motif of the LGN protein is responsible for directly binding to NuMA and recruiting Gαi. This Gαi-LGN-NuMA ternary spindle assembly complex then functions to anchor dynein motor proteins and control aster microtubule distribution, ultimately regulating spindle orientation. SAPCD2 disrupts this process by binding to the TPR motif of LGN and preventing association with Gαi at the apical pole 18. LGN and NuMA instead localize to the lateral poles of the cell and re-orient the spindle machinery, leading to a horizontal, symmetric cell division. The association of SAPCD2 with the Gαi-LGN-NuMA complex was demonstrated with mass spectrometry experiments. Immunoprecipitation (IP) experiments were conducted to determine whether the interaction between SAPCD2 and the spindle assembly complex is direct or indirect, and the authors reported that SAPCD2 was co-purified with LGN 18. Furthermore, recombinant LGN was utilized to successfully precipitate SAPCD2 from cell lysates, and NuMA inhibited SAPCD2 binding to the TPR motif in a dose-dependent manner 18. The spatiotemporal localization of SAPCD2 was explored, and biological experiments demonstrated undetectable levels of SAPCD2 in vertically diving RPCs while SAPCD2 localization was dependent upon mitotic state in horizontally dividing RPCs. In prophase and metaphase, SAPCD2 was strongly concentrated at the apical pole, but was barely detectable in anaphase and telophase, providing strong support for the role of SAPCD2 in orienting the cellular scaffolds during division. Furthermore, through the use of SAPCD2 knockout mice, this group made a compelling demonstration that SAPCD2 function is required for horizontal terminal cell divisions in murine RPCs, and it promotes symmetric terminal divisions generating two photoreceptor progeny cells 18.

In addition to regulating cellular scaffold assembly during mitosis, SAPCD2 has also been reported to participate in the Wnt/β-catenin pathway 19, further corroborating the potential role of SAPCD2 in coordinating pluripotency and cell fate specifications throughout development 19. In the Wnt/β-catenin pathway, β-catenin functions as a transcription factor that facilitates the transcription of several Wnt target genes (**Figure 2B**). Unphosphorylated β-catenin is transported to the nucleus and activates the transcription events by binding to the transcription complex upstream of the Wnt target genes, whereas phosphorylation of β-catenin inactivates its transcription activity. The phosphorylation β-catenin is catalyzed by the cytoplasmic protein complex composed of Axin1, APC, casein kinase 1 (CK1), and glycogen synthase kinase 3 (GSK3). Protein phosphatases, such as protein phosphatase 2A (PP2A), can act as antagonists to CK1 and GS3 by blocking the phosphorylation of β-catenin and maintaining the transcription of Wnt target genes. SAPCD2 functions to negatively regulate the transcription of Wnt target genes by outcompeting PP2A for binding to Axin1, reinforcing β-catenin phosphorylation. The direct interaction between SAPCD2 and Axin1 was confirmed by co-immunoprecipitation (co-IP) and GST pulldown experiments, and a series of SAPCD2 truncations co-transfected with Axin1 revealed that amino acids 1-239 of SAPCD2 are responsible for direct binding to Axin1 **(Figure 1 legend)**. Further *in vitro* and *in vivo* experiments demonstrated that Wnt3A-induced transcriptional upregulation was significantly reduced by SAPCD2 overexpression, and zebrafish embryos with reduced expression levels of SAPCD2 displayed a greater sensitivity to heightened Wnt activation. Importantly, Wnt3A activation increased protein levels of both β-catenin and SAPCD2, indicating that the transcriptional control between SAPCD2 and Wnt is bidirectional. Although the function of SAPCD2 in negatively regulating Wnt signaling appears to contradict its apparent role in tumorigenesis, other Wnt inhibitors have been found to be upregulated in cancers and function as oncogenic factors. For example, DKK-4 is an upstream inhibitor of Wnt signaling that promotes the migration and invasion of neoplastic cells and is aberrantly upregulated in colon cancer 20. The relationship between Wnt regulation and oncogenesis is certainly complex and requires further investigation.

## 3. The Role of SAPCD2 in Tumorigenesis: the Mechanisms and the Potential Clinical Applications

**Figure 3:**
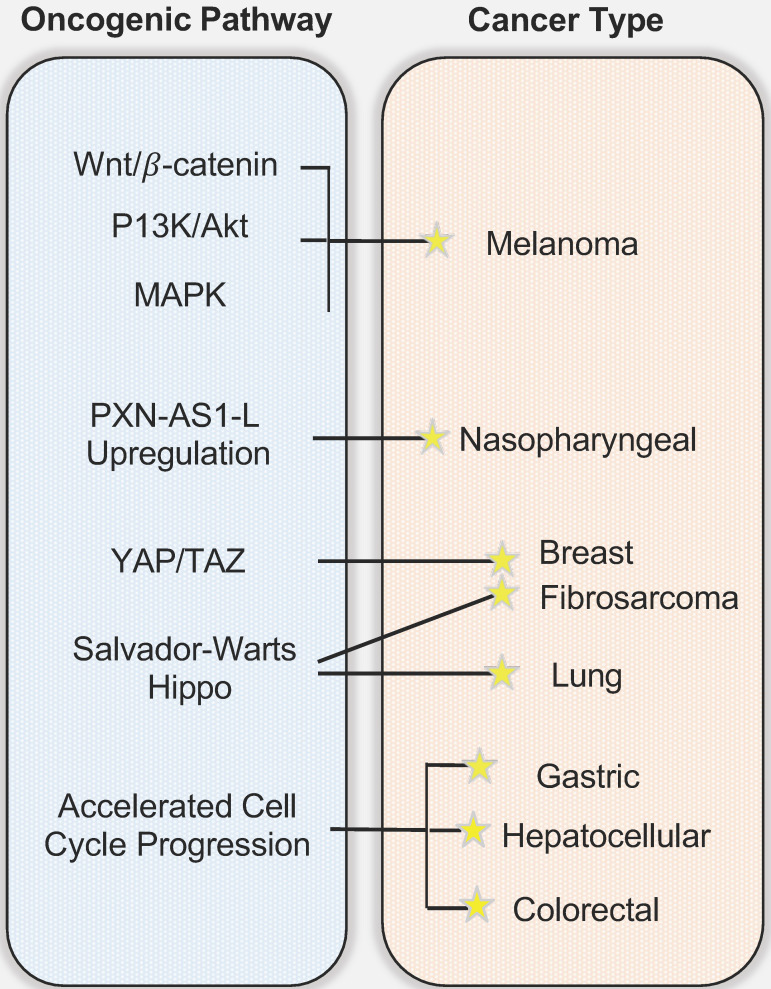
** The involvement of SAPCD2 in Tumorigenesis and the underlying mechanisms.** While the figure depicts the mechanisms/pathways that have been characterized in each specific cancer type, none of the illustrated oncogenic mechanisms are necessarily cancer-type specific and mutually exclusive, and it is likely that each cancer type arises from a combination of multiple pathways.

As reviewed above, SAPCD2 protein is highly expressed in various embryonic tissues, but its expression is normally undetectable after birth. The restored expression of SAPCD2 has been noted in several types of adult malignancies, suggesting that it plays an oncogenic role when overexpressed. Indeed, as reviewed in this section and summarized in **Figure 3**, recent studies have strongly supported that SAPCD2 is a key oncogenic factor that might be involved in the malignant transformation and metastasis of many types of cancers. The mechanisms by which SAPCD2 regulates key oncogenic cellular processes, including epithelial-mesenchymal transition (EMT), chromosomal instability and DNA damage responses, are summarized into **Figure 2,** together with its mechanisms of action in regulating normal cellular processes.

### 3.1. SAPCD2 in gastric cancer (GC)

SAPCD2 was originally identified in 2007 by Xu *et al*. as a novel oncogene contributing to GC 1. This group was the first to isolate the SAPCD2 gene in GC cell lines 1. They further found that SAPCD2 is specifically expressed in primary GC tissues but not in matched normal stomach mucosa 1. The group demonstrated that GC cells depleted of SAPCD2 expression exhibited a marked decrease in proliferative capacity in addition to G_2_/M phase cell cycle arrest 1. Furthermore, SAPCD2 depletion resulted in stalled tumor enlargement in a mouse GC xenograft model 1. From these results, it was concluded that SAPCD2 enhances tumorigenicity by aggravating cellular proliferation and malignant transformation 1. Since the original report, studies from other groups have further supported the role of SAPCD2 in GC. Cui *et al.* confirmed the differential expression of SAPCD2 in human GC when investigating the regulation of SAPCD2 expression by miR-29a 4. They found that SAPCD2 was upregulated in five of six tested human GC cell lines as well as 35 of 60 tested GC tissue samples 4. Additionally, siRNA-mediated knockdown of SAPCD2 *in vitro* inhibited GC cell proliferation and blocked cell cycle progression at the G_1_ phase 4. The tumorigenic role of SAPCD2 in GC was further reinforced by investigations conducted by Cao *et al* 21. The basal expression levels of SAPCD2 were demonstrated to be significantly higher in four human GC cell lines compared to the normal gastric epithelium cell line GES-1. The siRNA-mediated knockdown of SAPCD2 reduced cell proliferation in a GC cell line AGS1, while exogenous overexpression of SAPCD2 in GES-1 cells promoted cell proliferation, supporting the growth promoting function of SAPCD2. The oncogenic properties of SAPCD2 in GC cells were further demonstrated by wound healing and cell migration assays, which revealed a potent stimulatory effect by SAPCD2 on cell migration and invasion 21. The knockdown of SAPCD2 expression in AGS1 cells was accompanied by expression changes in E-cadherin, N-cadherin, and p-ERK protein levels 21. In addition to the *in vitro* experiments, this group further analyzed the SAPCD2 expression patterns in GC tissue samples. The results revealed an upregulation of SAPCD2 expression level with cytoplasmic localization in malignant gastric epithelium compared to tumor-adjacent normal tissues 21. The above results altogether demonstrate that the overexpression of SAPCD2 *in vitro* promotes proliferation, migration, and invasion of human GC cell lines as previously reported 21.

### 3.2. SAPCD2 in other cancer types

In addition to its function as an oncogene in gastric cancer, SAPCD2 has also been identified as a potential oncogene in several other cancer types. Jung *Y et al*. identified SAPCD2 gene as one of the 9 high confidence biomarkers of CRC 22. Another study reported that SAPCD2 is overexpressed at both the mRNA and protein levels in CRC specimens compared to matched normal mucosa and that high SAPCD2 mRNA and protein expression levels are directly correlated with poor tumor differentiation and poor post-surgical prognosis of CRC patients 23. Consistent with this finding, Luo Y *et al.* demonstrated that the protein expression level of SAPCD2 was significantly elevated in CRC tissues compared to adenoma and normal epithelial tissues as measured by immunohistochemical staining 17. They further showed that SAPCD2 functions to promote CRC cell proliferation, migration, and invasion *in vitro* and *in vivo* 17. Overall, the above studies support the oncogenic function of SAPCD2 in CRC.

SAPCD2 has also been identified as a potential driving factor in the tumorigenicity of hepatocellular carcinoma (HCC). Sun W *et al.* reported that the SAPCD2 protein is differentially expressed in primary hepatocellular carcinoma tumors and human HCC cell lines and observed a significant correlation between SAPCD2 expression level and tumor differentiation in HCC 24. Furthermore, SAPCD2 expression level is positively associated with enhanced HCC cell growth and colony formation *in vitro*, a finding that was corroborated by a measurable increase in tumorigenicity *in vivo* in a HEPG2 xenograft mouse 24. No significant relationship was established between SAPCD2 expression level and other factors contributing to HCC, such as viral Hepatitis B status 24, suggesting that SAPCD2 expression is an independent oncogenic factor. Additionally, enhanced SAPCD2 levels were found to upregulate expression of proliferating cell nuclear antigen (PCNA), cyclin B1, and mitotic arrest deficient 2 (MAD2), suggesting that the oncogenic function of SAPCD2 is related to its function in modulating cell cycle progression 24. The authors further showed that nearly 70% of screened HCC cells were positive for SAPCD2 expression 24, suggesting that SAPCD2 might be an oncogenic driver in a large fraction of HCC cases. Interestingly, the authors reported that 30% of healthy tumor-adjacent tissues were also positive for SAPCD2 expression 24, raising the possibility that SAPCD2 may only be oncogenic when its expression level exceeds a certain threshold. On the other hand, the expression of SAPCD2 in normal tumor-adjacent cells could potentially serve as an early indication of metastasis, but this is a speculative explanation and warrants further investigation through biological experiments.

SAPCD2 has also been associated with gliomas and melanoma. Wan W *et al.* found that SAPCD2 is overexpressed in some glioma tumor tissues, in which it is positively correlated with the degree of glioma malignancy, suggesting that SAPCD2 expression level may be a useful biomarker to grade gliomas tumors 25. In addition, the cellular localization of SAPCD2 expression differs between astrocytomas and glioblastomas, with SAPCD2 expressed in both the cytoplasm and the nucleus in astrocytomas while appearing exclusively in the cytoplasm in glioblastomas 25. The biological implications of this differential cellular localization, however, remain to be explored. The functions of SAPCD2 in promoting cell proliferation and invasion in melanoma and renal cell carcinoma (RCC) were also observed 26, 27. An interesting finding in melanoma reveals that a DNA vaccine targeting the SAPCD2 gene demonstrated a potential therapeutic effect on melanoma progression in a melanoma mouse model 28. This therapeutic strategy of targeting SAPCD2 is certainly worth further pursuit in other cancer types in the future.

A more recent study revealed the role of SAPCD2 in breast cancer 29. SAPCD2 expression levels were elevated in 48 human breast cancer tissue samples and was positively correlated with a number of prognostic indicators including tumor size, TNM stage, and lymph node metastasis, and negatively correlated with patient survival as revealed by Kaplan-Meier analysis 29, suggesting that SAPCD2 may be a useful prognostic indicator in breast cancer 29. They further show that depletion of SAPCD2 expression levels *in vitro* decreased breast cancer cell viability, migration, and invasion 29. In the investigation of the mechanisms of action of SAPCD2, it was found that SAPCD2 promotes the progression of breast cancer by stimulating expression of Yes-associated protein (YAP), an oncoprotein serving as a transcriptional co-activator with TAZ in YAP/TAZ signaling 29. The YAP/TAZ complex participates in the Hippo signaling pathway (also called the Salvador-Warts-Hippo Pathway) and is implicated in various physiological processes including the regulation of organ development and regeneration, embryonic growth, and wound healing 30. Elevated expression levels of YAP/TAZ or its nuclear translocation have been correlated with enhanced tumorigenicity in breast cancer where it functions to sustain tumor growth and promote metastasis 31.

SAPCD2 expression has been associated with various malignancies of the lung, including lung adenocarcinoma (LUAD), lung squamous cell carcinoma (LUSC), and non-small cell lung cancer (NSCLC) 32. Zhang X. *et al.* demonstrated through tissue microarrays that SAPCD2 expression is upregulated in both LUAD and LUSC compared to normal lung tissues 32. The expression level of SAPCD2 in LUSC was 17.8-fold higher than in matched normal mucosa, and expression in LUAD was 13.5-fold higher than matched normal mucosa. They further found that high SAPCD2 expression level is correlated with poor tumor differentiation and advanced histological grade in both LUSC and LUAD, supporting a role in tumor differentiation.

The expression of SAPCD2 in fibrosarcoma was demonstrated for the first time by Zhu *et al.* 33. Fibrosarcoma is a highly malignant cancer of mesenchymal origin with a high potential to metastasize to the lung. Interested in the relevance of SAPCD2 in fibrosarcoma and lung metastasis, Zhu *et al.* utilized both retrospective clinical investigations and *in vitro* experiments to investigate the role of SAPCD2 in aggravating fibrosarcoma 33. Kaplan-Meier analysis revealed that fibrosarcoma patients exhibiting a higher expression level of SAPCD2 are correlated with worse overall survival. Experimental investigations revealed that silencing SAPCD2 expression inhibits proliferation and induces apoptosis in human fibrosarcoma cell lines HT-1080 and SW684 and reduces lung metastasis *in vivo in a murine* subcutaneous fibrosarcoma xenograft model. Similar to findings in breast cancer, the inhibitory effects of silencing SAPCD2 on colony formation and anchorage-independent growth capabilities were reversed by constitutively active YAP1-S127A. The authors found that *in vitro* silencing of SAPCD2 led to increased expression of phosphorylated Hippo proteins p-MST1/2, p-LATS1, p-YAP1, and decreased nuclear translocation of YAP and TAZ. Furthermore, decreased SAPCD2 expression led to decreased expression of multiple other downstream genes in the Hippo pathway including CTGF, CYR61, SOX9, HOXA1, RPL13A, and PP1A at mRNA levels in fibrosarcoma cells, reinforcing the function of SAPCD2 in modulating expression of downstream Hippo signaling genes.

The function of SAPCD2 in pediatric cancer has also been proposed. A high maternal BMI is found to alter the methylation patterns of 20 CpG sites annotated to several genes, including SAPCD2, that are critical to cancer and cardiovascular diseases in infants 34. Together with the demonstrated role of SADPCD2 in adult cancers, this finding suggests that aberrant expression of SAPCD2 caused by epigenetic modulation of the SAPCD2 gene might be an important risk factor of cancer development in children born to mothers with high gestational body mass index (BMI) 34. The investigation of SAPCD2 in pediatric cancers is still at preliminary stage overall, and future studies are needed to further characterize its role in pediatric cancers.

### 3.3. The oncogenic mechanisms of SAPCD2

As reviewed above, the mechanisms underlying the oncogenic functions have been explored in multiple types of cancers along with the investigation of its oncogenic function in these cancer types. Except the function of SAPCD2 in regulating cell cycle distribution, which is believed to be one of the key mechanisms to contribute to its oncogenic function, there is lack of consensus whether other oncogenic mechanisms are universal or cancer type specific, which needs to be further investigated in future studies.

Interestingly, Mao L *et al.* utilized the fibroblast cell line NIH3T3 to investigate the oncogenic-promoting function of SAPCD2 15. The roles of SAPCD2 in altering cell cycle redistribution and regulating mitotic spindle assembly was demonstrated *in vitro* utilizing an exogenous SAPCD2 expression vector, which promoted mitotic progression, chromosomal missegregation, and ultimately, malignant transformation of NIH3T3 cells 15. Growth curve and cell proliferation experiments confirmed the proliferative capability of SAPCD2 and demonstrated that SAPCD2 confers anchorage-independent growth *in vitro*. These findings were expanded upon by an *in vivo* tumor model in which BALB/c nude mice injected with NIH3T3 overexpressing SAPCD2 developed tumors with typical histological features of fibrosarcoma 15. Aside from the observed impact of SAPCD2 on cell cycle progression and on the expression of a group of cell cycle proteins (reviewed above), this research group further demonstrated that SAPCD2 overexpression induces an increased expression level of γ-H2A.X in NIH3T3 cells 15, a biomarker for DNA double stranded breaks 35. While the presence of γ-H2A.X induces the activation of DNA damage repair pathways, the association of SAPCD2 expression with such an important biomarker for DNA damage indicates that chromosomal instability may be an additional mechanism by which SAPCD2 induces malignant transformation. This study clearly demonstrates the ability of SAPCD2 to promote the oncogenic transformation of non-cancer cells and provides insight into the oncogenic mechanisms of SAPCD2 in general in cancers.

Asides from the above *in vitro* investigations, *in silico* approaches have been utilized to predict the potential signaling network modulated by SAPCD2. In their effort to predict the SAPCD2 function, Zhang *et al.* first performed BLAST sequence analysis, but no obvious homology between SAPCD2 and any amino acid sequence of known function was revealed 16. They then utilized the predicted 3-dimensional (3D) structure of the EF-hand motif and the coiled coil domain in the SAPCD2 protein and predicted the potential protein regulatory network modulated by p42.3 by making structural similarity comparisons to proteins of known function. From such analysis, a selection of proteins with varying degrees of structural similarity to these domains in SAPCD2 was identified 16. This group further utilized Bayesian network analysis to predict the most likely tumorigenic pathway activated by SAPCD2, and identified the Ras pathway (i.e., Ras 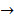 Raf-1 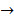 MEK 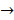 MAPK Kinase 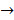 MAPK 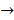 tubulin 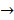 spindle protein 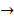 centromere protein 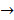 cell proliferation) as the most possible pathway activated by SAPCD2 16. Later on, this group identified an additional pathway possibly activated by SAPCD2 (i.e., S100A11 → RAGE → P38 → MAPK → tubulin → spindle protein → centromere protein → cell proliferation) after adjusting the similarity algorithm and optimizing the Bayesian network 7. The predictions provide an important direction in elucidating the mechanisms of action of SAPCD2, but rigorous experimental investigation is required to validate it.

Utilizing the same structural homology and functional similarity methods described above, another group predicted a pathway through which SAPCD2 regulates apoptosis, the FKBP → Bcl-2 → Bax → caspase-9 → caspase-3 → cell apoptosis pathway 36. Much like the pathways discussed above, this bioinformatics-predicted cellular network provides an interesting direction for future investigation but remains to be validated through vigorous *in vitro* and *in vivo* biological experiments.

### 3.4. The role of SAPCD2 in tumor metastasis and the underlying mechanisms

The potential role of SAPCD2 in driving tumorigenic metastasis has been indicated in several types of human cancer. Some of the findings on its role in metastasis are reviewed above as integral part of the investigation of its oncogenic function. This separate section is meant to summarize the findings on the mechanisms underlying its function as a modulator of tumor invasion and metastasis. Liu *et al.* demonstrated that SAPCD2 knockdown inhibits proliferation, migration, and invasion in melanoma cells while influencing both β-catenin expression levels as well as the P13K/Akt and MAPK pathways, which are known to play key roles in tumor metastasis 26. Li *et al.* revealed that knockdown of SAPCD2 expression in RCC cells inhibited cell proliferation and invasion 27, and they demonstrated that SAPCD2 knockdown in RCC cells significantly stimulated the expression of E-cadherin, repressed the expression of N-cadherin, and decreased the activation of β-catenin *in vitro* 27. A study in GC cell lines revealed that overexpression of SAPCD2 enhanced cell proliferation, migration, and invasion abilities, and this is coupled with reduced E-cadherin protein level and increased β-catenin and p-ERK proteins 21. The transition from high expression levels of E-cadherin to low expression levels of N-cadherin are trademarks of aggressive tumor growth and stimulates initiation of the EMT, a key process during the initiation of tumor metastasis. Zhu *et al.* additionally identified SAPCD2 overexpression being significantly correlated with metastasis in fibrosarcoma patients and demonstrated that SAPCD2 knockdown attenuated lung metastasis *in vivo* by activating the Hippo signaling pathway as described above 33. Together, the above reports illustrate the ability of SAPCD2 to influence the main metastatic signaling cascades.

### 3.5. The potential clinical applications of SAPCD2 in cancer diagnosis and therapeutics

As reviewed above, SAPCD2 expression at the mRNA and proteins levels has been found to be significantly elevated in a variety of cancer types; these findings were based on comparing cancer cell lines with immortalized normal cell lines 1, 4, 16, 21 or comparing tumor specimens with normal tissue specimens directly obtained from cancer patients 4, 17, 21, 23. These findings not only supported its important role in tumorigenesis, but also strongly suggested the potential to utilize SAPCD2 as biomarker for cancer diagnosis. Indeed, in an effort to identify high confidence biomarkers for CRC, Jung Y *et al*. analyzed 91 CRC candidate biomarker genes and identified SAPCD2 as one of the 9 high confidence biomarkers for CRC diagnosis 22, further supporting the potential value of SAPCD2 a key biomarker for the detection of malignancy. The potential of SAPCD2 as a prognostic marker for cancer progression have also been investigated. These studies have also been reviewed in detail as an integral part of the above subsections (*3.1* to *3.4*). To summarize, these studies revealed that higher expression levels of SAPCD2 in tumor specimens were correlated with poorer patient survival 29, 33, and that elevated SAPCD2 expression levels were positively correlated with a number of prognostic markers, including higher tumor stage, higher tumor size, higher histological degree of malignancy, worse tumor differentiation, and metastasis 24, 25, 29, 33. However, all the above findings were based on retrospective investigations. The reliability of SAPCD2 as biomarker for cancer diagnosis and prognosis certainly need to be further evaluated in perspective clinical investigations.

The important role of SAPCD2 in tumorigenesis also suggests that targeting SAPCD2 may be an effective approach to treat cancers. Indeed, siRNAs against SAPCD2 have shown therapeutic potential *in vitro* in cancer cell lines and *in vivo* in tumor xenograft models 1, 4, 21, 26, 29, 33. siRNAs against specific disease-driving genes have been explored as therapeutic agents to treat human diseases and clinical trials have shown promising results 37, 38. These findings on therapeutic siRNAs shed light on the development of therapeutic SAPCD2-targeting siRNAs for treating cancers, especially cancers with highly elevated SAPCD2 expressions. Besides using siRNA to target SAPCD2, a DNA vaccine targeting the SAPCD2 gene has been explored and exhibited therapeutic activity on melanoma progression in a melanoma mouse model 28. These preliminary investigations altogether demonstrated the potential of targeting SAPCD2 for cancer treatment. In order to translate these findings into clinical applications, however, extensive *in vivo* and preclinical investigations need to be conducted to further evaluate the therapeutic efficacy and toxicity of these SAPCD2-targeting agents.

## 4. Other Diseases Associated with SAPCD2

In addition to its role in cancers, some evidence in favor of SAPCD2 contributing to other diseases has emerged. For example, a study demonstrated that SAPCD2 expression is positively correlated with gastric mucosal inflammation and is enhanced by tumor necrosis factor (TNF)-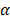 and H. pylori infection 39. Given the diverse protein interactions and biological functions of SAPCD2 as reviewed above, it is highly likely that aberrant expression and abnormal functions of SAPCD2 are involved in additional disease types aside from inflammation and tumorigenesis. Future studies are certainly warranted to better define its role in additional disease processes.

## 5. Regulation of SAPCD2 Expression and Function

Mechanisms that regulate the expression of SAPCD2 have been investigated, and several regulatory mechanisms have been characterized or predicted. Several studies showed that the aberrant SAPCD2 expression levels are associated with genetic and epigenetic alterations of the SAPCD2 gene, suggesting that the expression level of SAPCD2 is modulated at both the genetic and epigenetic levels. Zhang X. *et al.* investigated the genetic and epigenetic alterations of SAPCD2 in different subtypes of lung cancers 32. They found that LUSC samples demonstrated a lower level of SAPCD2 DNA methylation and a higher level of DNA amplification compared to LUAD. These differences between LUAD and LUSC, along with their phenotypic consequences, suggest that dysregulation of the SAPCD2 gene is likely controlled, in part, by gene amplification and epigenetic changes in lung cancer. Another study identified a single non-synonymous single nucleotide polymorphism (SNP) in the SAPCD2 gene in five lung cancer tumor samples, but not in normal lung mucosa samples 40. However, whether this SNP results in loss of function or gain of function of SAPCD2 and its resulting impact on lung cancer progression has yet to be explored.

**Figure 4:**
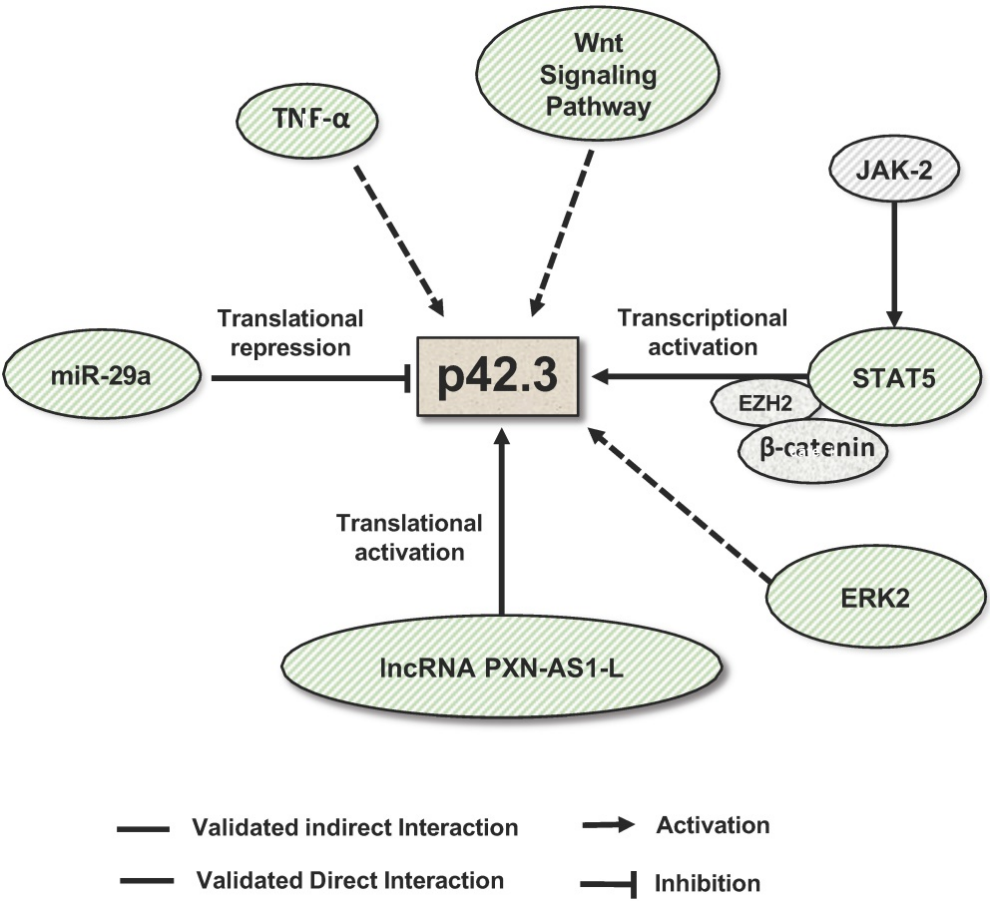
** Mechanisms that regulate SAPCD2 expression.** SAPCD2 expression was found to be regulated via multiple pathways. One of well-characterized pathways involves coordinated interactions of transcription factors STAT5, E2H2 and β-catenin. In addition, ERK2, TNF-α and Wnt signaling pathway are found to promote SAPCD2 expression. However, the detailed mechanisms underlying these regulatory pathways need to be further defined. Two non-coding RNAs are demonstrated to regulate SAPCD2 expression at translational level, with miRNA miR-29a repressing its expression and lncRNA PXN-As1-L promoting its expression.

The regulation of SAPCD2 expression at the transcriptional and post-transcriptional levels has also been investigated and several regulatory mechanisms have been experimentally validated (summarized in **Figure 4**). As reviewed above, SAPCD2 expression is regulated at the transcriptional level by a coordinated interaction of transcription factors STAT3, EZH2 and 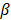-catenin, together activating the transcription of SAPCD2 3.

Like most protein-coding genes, SAPCD2 is predicted to be a direct target of miRNAs **(Table 1)**. miR-29a, a miRNA which functions as a tumor suppressor in multiple types of cancers 41, 42, is the first miRNA that was experimentally validated to bind to the SAPCD2 3'UTR and regulate SAPCD2 protein expression 4. An inverse relationship between miR-29a and SAPCD2 expression has been observed in GC cell lines as well as GC tissue samples. Furthermore, silencing SAPCD2 expression by miR-29a in GC cells resulted in G_1_ phase cell cycle arrest 4. These findings strongly suggest the biological relevance of miR-29a as a regulator of SAPCD2 function. On the other hand, the interaction of miR-29a with SAPCD2 also indicates that SAPCD2 is among a downstream effector of miR-29a that mediates its tumor suppressive function.

Another mechanism to modulate SAPCD2 expression is through long noncoding RNA (lncRNA). Like miR-29a, lncRNA PXN-AS1-L directly interacts with the 3'UTR of SAPCD2 mRNA 5. Contrary to the functions of miRNAs, PXN-AS1-L functions to increase the expression of SAPCD2 at the mRNA and protein levels 5. PXN-AS1-L expression levels are positively correlated with SAPCD2 expression levels 5, and both the expression levels of PXN-AS1-L and SAPCD2 are negatively associated with mean overall survival of NPC patients 5, supporting the clinical significance of the regulatory interaction of PXN-AS1-L with SAPCD2. The authors demonstrated the direct interaction between PXN-AS1-L and the 3'UTR of SAPCD2 by RNA pulldown assays. Interestingly, they further found that PXN-AS1-L overexpression reduced the association of AGO with the 3'UTR. This finding suggests that PXN-AS1-L is likely to upregulate SAPCD2 expression by preventing the miRNA binding to the target sites in the 3'UTR, and thereby releasing the SAPCD2 mRNA from the repression by miRNAs.

Additionally, multiple signaling pathways are found to regulate SAPCD2 expression. It was found that inhibition of the JAK/STAT, MAPK, and Wnt signaling pathways effectively downregulated the expression of SAPCD2 2. Overall, current knowledge suggests that SAPCD2 expression and function are regulated at all levels of genetic, epigenetic, transcriptional, translational and post-translational modifications.

## Conclusions

In summary, since the discovery of SAPCD2 as a cell cycle related gene in 2007, it has been intensively investigated and significant knowledge has been gained regarding its cellular functions, its mechanisms of action, and its relevance to human diseases. Findings from different research groups consistently demonstrate that SAPCD2 primarily functions as a mitotic phase-promoting factor by interacting with multiple proteins in the cell cycle interplay network. The SAPCD2 protein has also been shown to play an important role in determining cell polarization and cell fate throughout development. Not surprisingly, its function as a regulator of cell cycle progression contributes to its role in modulating cell fate. Additional mechanisms that contribute to its role in development have been discovered, such as its interaction with the Gαi-LGN-NuMA spindle assembly complex and the Wnt/β-catenin signaling pathway. Informatics analyses based on protein structure similarity have predicted additional mechanisms underlying its cellular functions. Further investigations on these predictions will help to elucidate its mechanisms of actions and may reveal additional functions of SAPCD2. The cellular functions summarized above have unsurprisingly underlined the role of SAPCD2 in tumorigenesis when aberrantly overexpressed in adult tissues. Indeed, the oncogenic function of SAPCD2 has been validated in *in vitro* and *in vivo* investigations and uncovered its potential as a valuable prognostic indicator of various types of cancers.

Despite the impressive advances that have been made to date, important questions remain to be answered. The role of SAPCD2 in certain cellular processes such as cell fate determination needs to be further characterized, and the signaling pathways underlying its expansive cellular functions requires further elucidation. As a novel oncogenic factor, the potential of developing SAPCD2-based diagnostic and therapeutic tools will certainly be an important direction in the field of cancer research. Given the critical oncogenic role that has been demonstrated in published studies, we believe that further research efforts will eventually benefit the treatment and health of cancer patients. Aside from its role in tumorigenesis, speculations on its role in other types of diseases, such as inflammation, certainly warrant future research effort as well.

## Figures and Tables

**Table 1:** ** miRNAs that are predicted to target the 3'UTR of SAPCD2 mRNA.** The miRNA:target prediction program TargetScan is applied to identify miRNAs that are predicted to target the 3'UTR of SAPCD2 mRNA. Shown are **(1)** names of SAPCD2-3'UTR:miRNA pairs, **(2)** sequences of the corresponding SAPCD2-3'UTR:miRNA pairs, and **(3)** the seed target sequence positions in the SAPCD2-3'UTR for the corresponding miRNAs.

(1) Name	(2) Target site:miRNA sequences		(3) Seed Position
SAPCD2 3' -UTR	5'	CUCCGUUUUGGCUCCUGGUGCUG 3'	214-220
hsa-miR-29a-3p	3'	UUGGCUAAAGUCU--ACCACGAU	
SAPCD2 3'-UTR	5'	GAUUGACUCUAGACCCAGAUA 3'	2447-2454
hsa-miR-1298-3p	3'	CAAGUCAGUCAACGGGUCUAC 5'	
SAPCD2 3'-UTR	5'	CCCACAGCAACCCCACAGAGCCA 3'	1350-1357
hsa-miR-760	3'	GGGUGUCUGG-----GUCUCGGC 5'	
SAPCD2 3'-UTR	5'	ACCCCCCUUUGCUCUCACGCCCA 3'	848-855
hsa-miR-1268a	3'	GGGGGUGGUG-----GUGCGGGC 5'	
SAPCD2 3'-UTR	5'	ACCCCCCUUUGCUCUCACGCCCA 3'	848-855
hsa-miR-1268b	3'	GUGGGGGUGGUG---GUGCGGGC 5'	
SAPCD2 3'-UTR	5'	CCCAGUUGAGAAUCUGCCCCA 3'	2167-2174
hsa-miR-486-3p	3'	UAGGACAUGACUCGACGGGGC 5'	
SAPCD2 3'-UTR	5'	GCCCUGGAGACCACGAGGGGA 3'	1906-1913
hsa-miR-6795-3p	3'	GACCCCCUUCUUUGCUCCCCA 5'	
SAPCD2 3'-UTR	5'	ACUGGACUGGAAGCAGGAGA 3'	1696-1703
hsa-miR-1976	3'	UGUCGUUCCUCCCGUCCUCC 5'	
SAPCD2 3'-UTR	5'	CAGGGCCAGCCU-GGCACUCA 3'	36-43
hsa-miR-1909-5p	3'	GUCCCGUCCGUGGCCGUGAGU 5'	
SAPCD2 3'-UTR	5'	AAAGCCUGUUCCCCCGACUCA 3'	798-805
hsa-miR-4785	3'	CGACCGCCGCAGCGGCUGAGA 5'	
SAPCD2 3'-UTR	5'	ACUUCCAACAACGGGCAGCAGA 3'	2419-2426
hsa-miR-3692-5p	3'	CAUAGGUGAGGACUGGUCGUCC 5'	

## Abbreviations

3D: 3-dimensional
3'UTR: 3' untranslated region
5'UTR: 5- untranslated region
aa: amino acid
BMI: body mass index
CC: coiled coil
CDC: cell division cycle
Cdc2-Tyr15: Cdc2 phosphorylated at position tyrosine 15
CDS: coding sequence
ChIP: chromatin immunoprecipitation
Chk2: checkpoint kinase 2
Ck1: casein kinase 1
co-IP: co-immunoprecipitation
CRC: colorectal cancer
EMT: epithelial-mesenchymal transition
GC: gastric cancer
GPSM2: G protein signaling modulator 2
Gsk3: glycogen synthase kinase 3
HCC: hepatocellular carcinoma
IP: immunoprecipitation
lncRNA: long non-coding RNA
LUAD: lung adenocarcinoma
LUSC: lung squamous cell carcinoma
MAD2: mitotic arrest deficient 2
MEK: mitogen-activated protein kinase
miRNAs: microRNAs
NPC: nasopharyngeal carcinoma
NSCLC: non-small cell lung cancer
nts: nucleotides
PCNA: proliferating cell nuclear antigen
PKC: protein kinase C
PP2A: protein phosphatase 2A
pSTAT5: phosphorylated STAT5
RCC: renal cell carcinoma
RPCs: retinal progenitor cells
SAPCD2: Suppressor APC Domain Containing 2
siRNA: short interfering RNA
SNP: single nucleotide polymorphism
TNF: tumor necrosis factor
TPR: tetratricopeptide
YAP: yes-associated protein
